# Baseline Assessment of Risk Factors of Presumptive Tuberculosis among under Five Children Living with an Index Client under Treatment in Mbale District, Eastern Uganda

**DOI:** 10.9734/mrji/2020/v30i530214

**Published:** 2020-06-02

**Authors:** Rosemary Tumusiime, Charles Mukasa, Agatha K Kisakya-Maria, Irene Mildred Neumbe, Jerome Odyeny, Bernard Maube, Yahaya Gavamukulya, Rebecca Nekaka

**Affiliations:** 1 Department of Community and Public Health, Faculty of Health Sciences, Busitema University, P.O.Box, 1460, Mbale, Uganda; 2 Busiu Health Center IV, Mbale District Local Government, Mbale District, Uganda; 3 Department of Biochemistry and Molecular Biology, Faculty of Health Sciences, Busitema University, P.O. Box, 1460, Mbale, Uganda

**Keywords:** Presumptive TB, under five children, Index clients, contact tracing, Mbale district

## Abstract

**Background and Aims:**

Children in contact with adults having pulmonary Tuberculosis (TB) are vulnerable to TB infection and hence contact tracing and screening is important for early detection of infection. However, there are few contacts traced and the prevalence and risk factors for transmission are not well studied. The objective of this study was to determine the prevalence of infection and risk factors associated with TB transmission among under five children in household contact with adult pulmonary TB patients.

**Materials and Methods:**

A cross sectional study was carried out in three health facilities with a high TB burden in Mbale District, Eastern Uganda involving all under five household contacts of adults with pulmonary tuberculosis recorded in the TB register from October 2018 to March 2019 and still on treatment. Structured questionnaires were administered to the index clients to obtain their demographic and clinical data about TB, HIV as well as information on the children. Children were screened using the intensive case finding forms to identify presumptive cases.

**Results:**

The total number of index TB Clients line listed were 70. Number of clients traced was 38, 21 (%) of whom had children under five years and a total of 33 children were identified. The number of presumptive cases was 9/33 (27.27%). 77.8% of the presumptive cases were living in poorly ventilated houses.

**Conclusion:**

The study identified children with presumptive TB and various risk factors for TB transmission. Intensive contact tracing can therefore help reduce TB transmission within the communities. It is recommended to undertake studies aiming at improving contact tracing and strategies to eliminate the risk factors to TB transmission.

## 1. INTRODUCTION

Tuberculosis (TB) remains one of the major causes of high morbidity and mortality accounting for 10.0 million new cases and 1.3 million deaths among HIV-negative individuals globally [1,2] In 2017, the World Health Organization (WHO) estimated that 10% of the 10 million tuberculosis (TB) incident cases occurred in children, which resulted into 210,000 TB related deaths(1). Children who are exposed to TB index cases, particularly sputum smear positive pulmonary TB(PTB), are at high risk of infection and when infected, the infants and young children (below 5 years) are more likely of developing the disease [2]. The risk of TB transmission increases with sputum positive index cases and the infectiousness directly depends on the concentration of bacilli in the sputum of a TB client [3]. Therefore, the risk of acquiring the disease increases with proximity and prolonged contact duration especially with untreated sputum positive patients.

Identifying and treating children with TB is challenging as the disease manifests itself differently in this age group compared to adults. Additionally, children with TB in their lungs have fewer TB bacteria making it nearly invisible to many common diagnostic methods. Finally, it is very hard to obtain sputum samples needed to perform the diagnostic tests in children due to difficulty in coughing. WHO recommends routine screening of child contacts in resource limited settings using a symptom-based screening approach that can be easily implemented in the community and provision of preventive therapy for at risk contacts after excluding TB [4]. The recommended regimen is isoniazid preventive therapy (IPT) that is provided as a daily dose for at least 6 months. Despite the potential benefits of contact screening for active case detection and initiation of IPT, these activities are rarely implemented in TB endemic settings [5,6].

Uganda is a TB endemic resource limited country, which registered 52,458(65%) new TB cases out of the expected 80,000 TB cases in 2017/2018 and 5,100(9%) were children <15 years [7]. Mbale District recorded a TB notification rate of 72% and 1,875 (60%) of these were confirmed bacteriological pulmonary TB cases [7]. TB contact tracing and screening are recommended by the National TB and leprosy control programme in Uganda but TB case detection strategy has been limited to passive screening.

The goal of TB control programs is to eliminate the disease by breaking the chain of transmission, and this can be effectively achieved through rapid identification and effective treatment of infectious individuals [2].

Contact tracing, a form of active case finding has gained importance and it is now incorporated in the revised National TB/leprosy control program of the government of Uganda [8,4]. For some time now, particular attention has been given to TB in children by Uganda’s NTP because TB treatment is recognized as an opportunity that prevents and addresses an important cause of child mortality [9]. Although guidelines for TB contact tracing have been in existence, implementation is still a big challenge. This may stem from uncertainty about the potential yield of this strategy when targeting specific categories of TB index cases. The objective of this study was to undertake baseline assessment of the risk factors associated with TB transmission among under five children (U5C) in household contact with adult pulmonary TB patients.

## 2. MATERIALS AND METHODS

### 2.1 Study Design

A cross sectional quantitative study was carried out.

### 2.2 The Study Area/ setting

The study was carried out in Mbale Regional Referral Hospital, Namatala Health Centre III (which are located in Mbale Municipality) and Busiu Health Centre IV (located in Busiu Town Council) in Mbale District. These are high volume sites (Health facilities with highest number of TB clients in the district). Mbale District is located in the mid- Eastern part of the country. The district has a population of approximately 488,960 people and a population density of 1,096 persons per square kilometer [10]. At 80 per 100,000/ year, Mbale had the lowest district level TB incidence in Uganda in 2018 [7].

### 2.3 Study Participants

Adults who had been diagnosed with pulmonary TB in the period of October 2018 to March 2019 at public clinics in the three facilities in the district (index clients) were consecutively asked to participate in the study. Index clients were eligible to participate if they were 18 years and above, had a recorded TB diagnosis based on bacteriological confirmation, had been a resident of Mbale district for at least 6months and consented to a home visit by the study team. A household contact was defined as any person living on the same residential plot who shared either the same house or frequent meals with the index client. Index clients who participated provided written informed consent and completed a survey that included demographic characteristics and HIV clinical history. TB diagnosis and treatment data for index client were abstracted from the TB clinic registers.

TB registers for the previous six months of October 2018 – March 2019 were reviewed at the various health facilities to identify and generate a list of bacteriologically confirmed TB clients who were on treatment, with help of the TB focal persons. Together with the village health teams, we got the addresses, phone contacts of the index clients and made appointments to visit their homes. All identified TB clients were traced.

### 2.4 Household Visits

The study staff visited index client’s households within one week of recruitment. Household contacts were eligible to participate in the study if they met the definition of a household contact and informed consent was obtained from the caretakers.

Trained study staff administered questionnaires to the caretakers and screened the children under five years for signs and symptoms of TB, and collected one sputum sample from each respondent for smear and culture in accordance with national guidelines [11]. All the under 5 household contacts were referred for further clinical assessment through routine services, including evaluation for eligibility for isoniazid preventive therapy, according to Uganda TB guidelines [2]. HIV testing was not provided but clients who did not know their HIV status were referred to the routine health services for voluntary counseling and testing.

### 2.5 Data Collection Methods

The interview structured questionnaires were administered to the index clients to obtain the demographic data and clinical data about TB, HIV for index clients and the U5C. The risk factors for Tuberculosis infection among children were also collected using the questionnaires. Children were screened using the intensive case finding (ICF) forms to identify presumptive cases. ICF forms are used to screen clients for TB, by taking history of the cardinal signs like: cough and fever lasting more than 2 weeks, weight loss or poor weight gain among children, and night sweats; and a patient is deemed presumptive if they say yes to any of them. They are the recommended screening tools for TB [12].

### 2.6 Data Management and Analysis

The questionnaire forms were collected after being filled and compiled. The data was double entered in Microsoft excel worksheets, cleaned and then exported to STATA software for management and statistical analysis using measures of central tendencies, and dispersion. P-values were determined. The filled questionnaires were securely and confidentially stored.

## 3. RESULTS AND DISCUSSION

### 3.1 Results

**Fig. 1.**
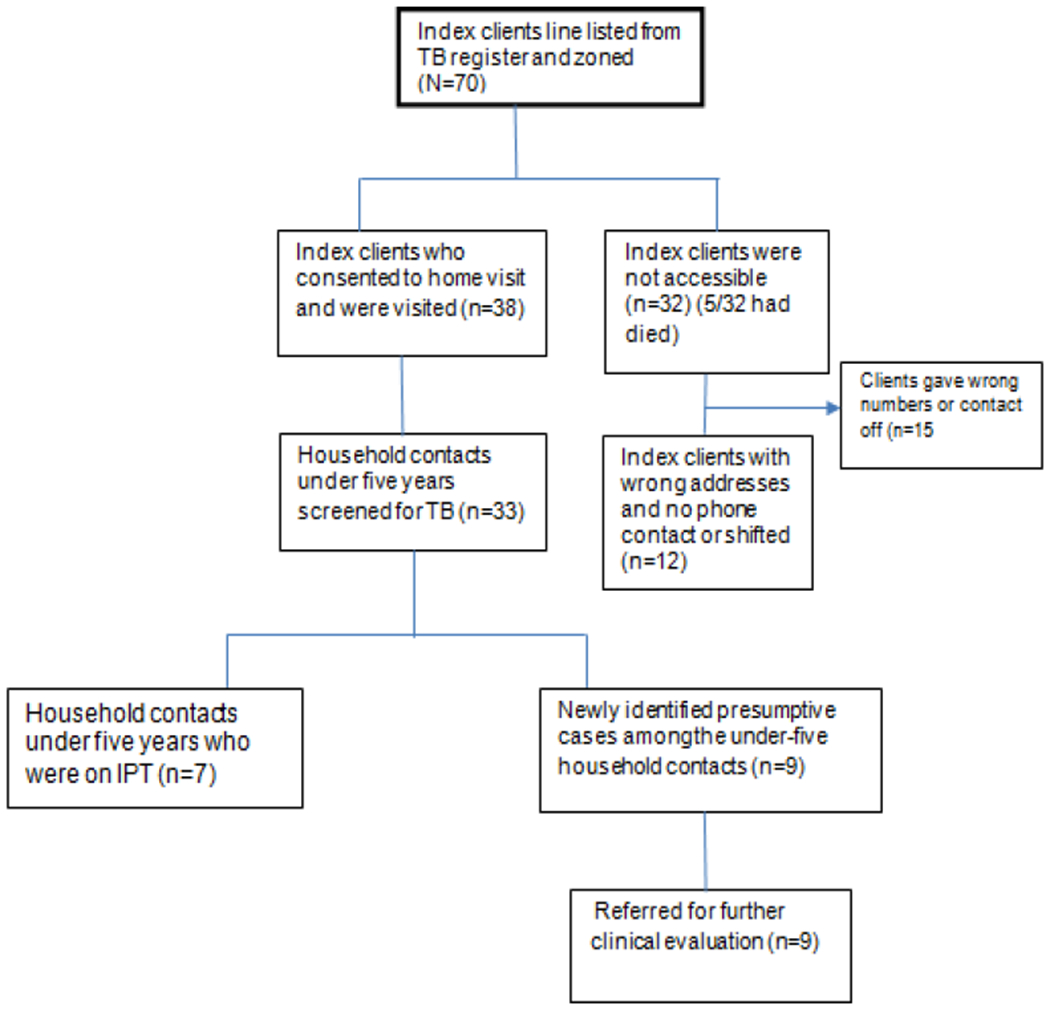
Flow chart showing summary of the study findings

From April 8^th^ 2019 to May 2 2019, we line listed and visited 38 (54%) of the 70 eligible index clients from three participating public health facilities in Mbale District (Fig. 1). Majority of the index clients 40 (57.1%) were getting treatment from Mbale Regional Referral Hospital, 19(27.1%) were from Namatala Health Centre IV and 11(15.8%) were from Busiu Health Centre IV.

### 3.2 Index Clients

Index clients were 65.8% male (25/38, 65.8%), female (13/38, 34.2%) and the mean age was34.5 years (Table 1). Only 13%of the index clients had completed at least some high school and over half (23/38, 61%) were not employed. Majority (28/38, 74%) of the households of index clients had semi-permanent and temporary housing and 23, (61%) of the houses were poorly ventilated. Nearly half of the clients were sharing rooms with their family members.

### 3.3 Household Contacts

From the 38 index clients visited, we screened 33 under five household contacts (Table 2). The mean age of the contacts was 3 years and almost half 18(52.9%) of the children were female. All the children had received BCG immunization at birth.

As shown in Table 3,the total number of index TB Clients line listed were 70, of these 40 (57.1%) from Mbale regional referral hospital (MRRH), 19(27.1%) from Namatala HCIV and 11(15.8%) from Busiu HCIV. Number of clients traced was 38, 21(%) of them had U5C and a total of 33 children were identified. The total number of presumptive TB cases was 9(%) and of the 33 children, 7(%) were on Isoniazid Preventive Treatment (IPT).

From the Table 4, among house type the semi-permanent houses 5(55.6%) of the children were found presumptive. With family size above 10 people presumptive TB children were 6(66.6%). In households where the contacts shared rooms with TB patients 4(44.4%) were presumptive. Homes with poor ventilation, 7(77.8%) of the children were found presumptive.

### 3.2 Discussion

The study found a high prevalence of presumptive TB cases (27.27%, 9 out of 33) among the contacts and all the nine children were referred to the health facility for further evaluation. All the other children who were asymptomatic and not on IPT were referred for initiation of IPT. In regard to screening of child contacts of TB patients, WHO recommends that only symptomatic children require referral to a level of care where appropriate assessment for suspected TB can be undertaken. This assessment may include TST, CXR and sputum examination [4]. The WHO recommends that child TB contacts aged <= 5 years or HIV infected children of any age) without any symptoms suggestive of TB should be started on preventive therapy [13]. We acknowledge that no cases in our study were microbiologically confirmed. This is due to the fact that most of the children were unable to produce sputum. Nevertheless, all cases underwent careful clinical assessment, including checking information from the interview with objective data such as the growth chart for reported weight loss. It was reported that the chances of a child developing TB can be reduced by 60% if given IPT [14], however, in the current study only 7/33(21.2%) of the children were on IPT. This is more than the national rate of 8.4% (7.7–9.2) [15]. IPT delivery is still a challenge in our economically disadvantaged society and despite the fact that we referred many of the contacts to the hospital, majority were unable to go due to lack of transport. If a patient cannot afford transport to the facility, there is almost no way they can access the services. Studies elsewhere have attributed low initiation rate of IPT to lack of leadership for preventive interventions such as IPT initiation, low awareness about IPT, lack of parents’ knowledge on its benefits and perceived toxicities of Isoniazid [16]. Challenges in excluding TB disease in child contacts partly explains the limited application of recommendations to use IPT in most resource limited settings.

Our results showed a positive association between presumptive TB and being a contact of a bacteriologically confirmed TB case. This is consistent with previous studies in which a correlation between the level of infectivity of the index case and infection among contacts was found [6,17]. Our results did not show a significant association between household size and presumptive TB prevalence. Nevertheless, we found that 66.7% of the contacts with presumptive TB had a family size of 10-15 family members. Overcrowding has been described elsewhere as a potential risk factor for TB transmission [18]. Findings from a study in South Africa found a higher estimated risk of TB transmission in households where the index clients had a larger family size of about 10-15 people [19]. It was also found that 55.6% of the contacts lived in households with semi-permanent housing and 77.8% had poorly ventilated houses. Findings from a study carried out on the impact of ventilation on TB transmission showed that poor ventilation led to increased TB transmission (78%) in poorly ventilated cells [20]. Lower socioeconomic status and poor housing conditions have been linked with a higher risk of progression to active disease along other socio-medical factors like malnutrition [21].

Limitations of the study included incomplete data in TB registers making it hard for some clients to be traced, Hiding information about location of the clients by communities, aggressiveness of some clients, high rate of stigma in the communities, hard-to-reach areas, and the inability to confirm TB in the children.

## 4. CONCLUSION

Household contact tracing of bacteriologically confirmed TB index clients feasibly identified a substantial number of under five children with presumptive TB. Symptom screening using the intensified case finding form was an effective strategy for identifying cases in the household. Household contact tracing is an important component of comprehensive strategies to end TB in rural high burden settings. Further studies with follow-up component are needed on clinical TB, sputum induction for children. Furthermore, programs addressing socio-economic challenges should be integrated into TB services.

## Figures and Tables

**Table 1.** Demographic findings of index clients

Demographic findings		Frequency (%)
AGE	9-29	15(39.5)
	30-49	18(47.4)
	50 and above	5 (13.2)

SEX	Female	13(34.2)
	Male	25(65.8)

EDUCATION LEVEL	No formal education	3(7.9)
	Primary	20(52.6)
	O-Level and above	15(39.5)

OCCUPATION	Employed	15(39.5)
	Unemployed	23(60.5)

TYPE OF HOUSING	Permanent	10(26.3)
	Semi-permanent	21(55.3)
	Temporary	7(18.4)

VENTILATION	Poor	23(60.5)
	Good	13(34.2)
	Excellent	2(5.3)

ROOM SHARING	No	21(55.3)
	Yes	17(44.7)

**Table 2.** Demographic data of the child contacts of the index clients

Characteristic		Frequency (%)
AGE	1-2	20(60.61%)
	3-5	13(39.39%)

Gender	Female	17(51.5%)
	Male	13(48.5%)

BCG VACCINE	YES	33(100%)
	NO	00 (0%)

IPT	No	31(81.6%)
	Yes	7(18.4%)

**Table 3.** Number of index clients and contacts outcome ineach facility

Facility	Total No (%)	Clients traced	Clients with U5C	No. of U5C	No. presumptive	No. on IPT
MRRH	40(57.1)	23(60.5)	11(52.4)	21(63.6)	9(100)	5(71.4)

NAMATALA HCIV	19(27.1)	9(23.7)	6(28.6)	7(21.2)	0(0)	0(0)

BUSIU HCIV	11(15.8)	6(15.8)	4(19.0)	5(15.2)	0(0)	2(28.6)

TOTAL	70(100)	38(100)	21(100)	33(100)	9(100)	7(100)

**Table 4.** Factors associated with tb transmission among house hold contacts

Demographic characteristics			
		Presumptive	P-value

House_ Type	NO	YES	
PERMANENT	8(33.3)	3(33.3)	**0.968**
SEMI-PERMANENT	14(58.3)	5(55.6)	
TEMPORARY	2(8.3)	1(11.1)	
FAMILY SIZE	NO	YES	0.473
0-5	9(45.0)	2(22.2)	
10-15	10(50.0)	6(66.7)	
16-20	1(5.0)	1(11.1)	
ROOM SHARED	NO	YES	
NO	10(41.7)	5(55.6)	0.475
YES	14(58.3)	4(44.4)	
VENTILATION	NO	YES	
POOR	8(33.3)	7(77.8)	0.063
GOOD	12(50.0)	2(22.2)	
Excellent	4(16.7)	0(0.0)	

## ABBREVIATIONS

COBERS: Community Based Education Research and Services
HCIV: Health Centre IV
TB: uberculosis
IPT: Isoniazid Preventive Therapy
ICF: Intensive Case Finding
U5C: Under Five Children
